# NMR Spectroscopic Studies of Cation Dynamics in Symmetrically-Substituted Imidazolium-Based Ionic Liquid Crystals

**DOI:** 10.3390/ijms21145024

**Published:** 2020-07-16

**Authors:** Debashis Majhi, Andrei V. Komolkin, Sergey V. Dvinskikh

**Affiliations:** 1 Department of Chemistry, KTH Royal Institute of Technology, SE-10044 Stockholm, Sweden; majhi@kth.se; 2 Faculty of Physics, Saint Petersburg State University, 199034 Saint Petersburg, Russia; a.komolkin@spbu.ru; 3 Laboratory of Biomolecular NMR, Saint Petersburg State University, 199034 Saint Petersburg, Russia

**Keywords:** ionic liquids, liquid crystals, ionic liquid crystals, molecular orientational order, NMR spectroscopy

## Abstract

Ionic liquid crystals (ILCs) present a new class of non-molecular soft materials with a unique combination of high ionic conductivity and anisotropy of physicochemical properties. Symmetrically-substituted long-chain imidazolium-based mesogenic ionic liquids exhibiting a smectic liquid crystalline phase were investigated by solid state NMR spectroscopy and computational methods. The aim of the study was to reveal the correlation between cation size and structure, local dynamics, and orientational order in the layered mesophase. The obtained experimental data are consistent with the model of a rod-shaped cation with the two chains aligned in opposite directions outward from the imidazolium core. The alignment of the core plane to the phase director and the restricted conformations of the chain segments were determined and compared to those in single-chain counterparts. The orientational order parameter *S*~0.5–0.6 of double-chain ionic liquid crystals is higher than that of corresponding single-chain analogues. This is compatible with the enhanced contribution of van der Waals forces to the stabilization of smectic layers. Increased orientational order for the material with Br^−^ counterions, which exhibit a smaller ionic radius and higher ability to form hydrogen bonds as compared to that of BF_4_^−^, also indicated a non-negligible influence of electrostatic and hydrogen bonding interactions. The enhanced rod-shape character and higher orientational order of symmetrically-substituted ILCs can offer additional opportunities in the design of self-assembling non-molecular materials.

## 1. Introduction

Ionic liquid crystals (ILCs) are nanostructural soft materials which combine the orientational ordering behavior of liquid crystals with the properties of ionic liquids (ILs) [1]. It is anticipated that these non-molecular compounds are going to have a strong impact on the future development of functional materials for a variety of applications. For example, ILC-based devices for low-dimensional transport of ions and electrons hold great potential for applications in electrochemistry [2,3]. ILCs have already been used as templates to synthesize mesostructured porous materials [4,5,6] and as orientationally-ordered ionic solvents in catalysis [7,8]. On a fundamental level, the long-range-ordered structures exhibited by ILCs offer a model environment to explore anisotropic dynamic properties of ions self-organized in two-dimensional (2D) and three-dimensional (3D) space. A better understanding of self-assembling and nanosegregation behavior of ILs contributes to improved designs towards the development of ILC-based devices for specific applications.

The imidazolium derivatives are the most popular cations for IL synthesis [9]. While the majority of studies have explored single-chain asymmetrically substituted imidazolium cores, there have been a number of recent reports on the investigation of imidazolium-based ILs with two symmetrically attached long alkyl chains [3,10,11,12,13,14,15]. Relocation of the polar and apolar domains provides a route to tune the physicochemical properties of ionic liquids while the enhanced rod-shape character of a symmetrically-substituted cation offers additional opportunities for self-assembly [13,16]. By increasing the chain length to 12 or more, double-chained ILs exhibit liquid-crystalline phases spanning a wide temperature range dependent on anion type. Symmetric ILCs give rise to smectic phases in agreement with the rod-shaped structure of cations. Detailed phase diagrams and thermodynamic parameters have been described [3,10,11,14,15,16]. Often, the temperature range of the smectic phase in symmetric ILCs is compared favorably to that of the single chain analogues. In contrast to conventional molecular thermotropics, ILCs have no bulky structurally rigid core. The extended length of the cation with symmetrically-arranged side chains can contribute more effectively to mesophase stabilization due to the increasing role of van der Waals forces inducing the orientational ordering of ions [17]. Other significant interactions in IL mesophase include electrostatic and hydrogen bond forces within the ionic sublayer which induce segregation of ionic constituents into distinct nanosized regions [1]. Further experimental and theoretical studies are needed to understand the delicate equilibrium and complex landscape of different inter-particle forces driving mesophase formation in ILs.

In this study, we use solid-state NMR spectroscopy to characterize the structural and dynamic properties of ILCs with the imidazolium cation symmetrically substituted with long alkyl chains at the atomic and molecular levels. NMR spectroscopy is a powerful experimental tool to non-invasively obtain high-resolution information on molecular structure and the mobility of materials in liquid-crystalline phases [18]. Anisotropic spin interactions, such as dipolar or quadrupolar couplings, have provided information on molecular ordering, director alignment, local dynamics, and conformation in ILCs [19,20,21,22,23]. Such a wide range of information in a single method is not possible to obtain with any other spectroscopic technique. Herein, we apply advanced one-dimensional (1D) and 2D solid-state NMR techniques to probe pair-wise dipolar spin couplings among ^1^H, ^13^C, and ^15^N spins. This technique offers direct access to so-called bond order parameters which comprehensively describe local dynamics. The large chemical shift dispersion of ^13^C and ^15^N spins contributes to spectral resolution and simplifies the assignment of dipolar splittings. A complementary approach is measuring the quadrupolar couplings of ^2^H spins. Natural abundance deuterium (NAD) NMR is an increasingly popular technique in liquid crystal research [24]. Recently, we have applied multinuclear NMR approaches to study ion dynamics in more conventional single chain ILCs [20,21,22]. In the present work, various NMR spectroscopic methods were combined to gain quantitative information on ionic mobility and contribute to our understanding of the fundamental processes of dynamics of ionic species in symmetrically-substituted ILCs. On the basis of experimentally determined large sets of the order parameters, we put forward models for the motion of organic cations in the smectic phase.

## 2. Results and Discussion

### 2.1. NMR Spectra

**Scheme 1:**
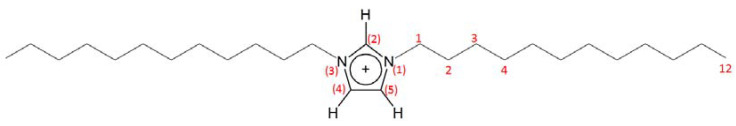
The structure and labelling schema of the C_12_C_12_im cation.

Two symmetric imidazolium-based ionic liquid crystals, bis-1,3-dodecyl imidazolium bromide (C_12_C_12_imBr) and tetrafluoroborate (C_12_C_12_imBF_4_), were studied. The materials exhibited smectic A phases in temperature ranges comparable to those of single-chain analogs and in agreement with reported phase diagrams (Table S1 in Supplementary Materials) [10,13,14,15,25]. Residual water content in the samples were estimated by examining the ^1^H NMR spectra of the neat samples in the isotropic phase (Table S1). Cation structure and atom labelling for referencing NMR spectra are shown in Scheme 1. NMR spectroscopic measurements in the smectic phase were performed in the samples with the director aligned perpendicular to the magnetic field. The sample alignment was achieved by slow cooling from the isotropic phase in the presence of a strong magnetic field *B*_0_ = 11.7 T.

**Figure 1:**
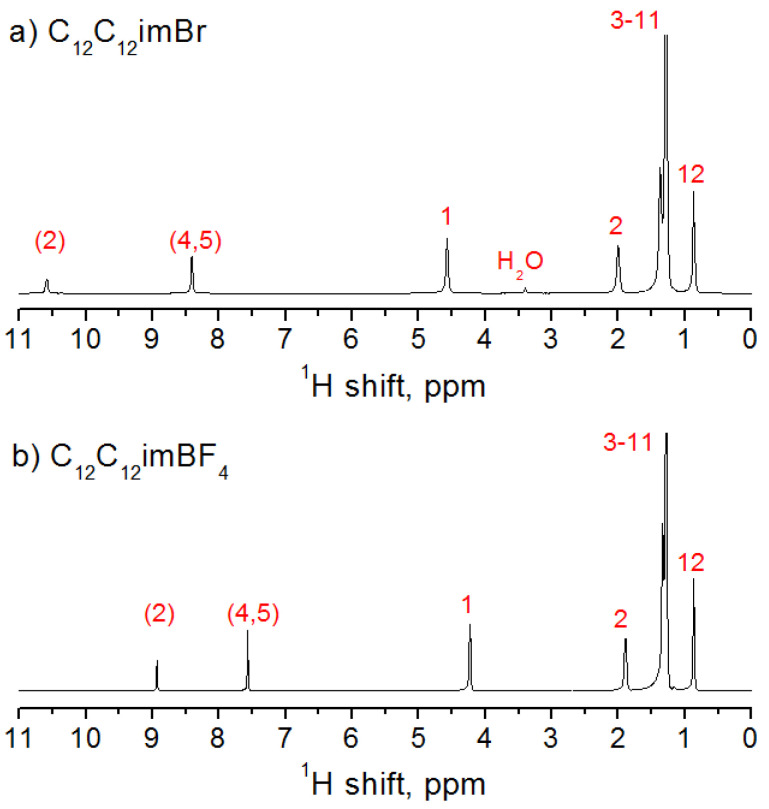
^1^H NMR spectra of C_12_C_12_imBr (**a**) and C_12_C_12_imBF_4_ (**b**) in the isotropic phase at 123 °C.

**Figure 2:**
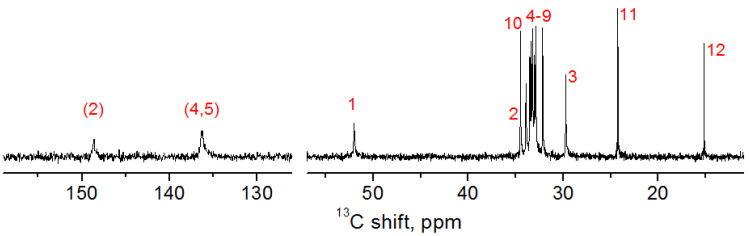
Carbon-13 cross polarization NMR spectrum of C_12_C_12_imBr in smectic A phase at 97 °C.

Proton spectra in the isotropic phase of neat ionic liquids are displayed in Figure 1. Compared to its BF_4_ counterpart, bromide salt exhibited a strong downfield shift of the imidazolium proton signals, in agreement with the higher ability of the Br anion to form a hydrogen bond [26,27]. In the smectic phase, proton spectra (not shown) were strongly broadened by homonuclear dipolar proton coupling and individual proton signals were not resolved. The representative ^13^C NMR spectrum of the C_12_C_12_im ions in the smectic A phase is shown in Figure 2. The assignment of the resolved carbon signals was performed by comparison to spectra of asymmetric analogues [19,20,21,22] and was confirmed by the multidimensional correlation spectra described below. The observed chemical shift (CS) was given by the component δ_⊥_ of the residual CS tensor [18,20]. The narrow carbon-13 resonances observed in the smectic phase under static sample conditions indicated well-defined director alignment with respect to the external magnetic field. High spectral resolution permitted detailed investigation of conformational dynamics of the organic cation by dipolar NMR spectroscopic methods.

**Figure 3:**
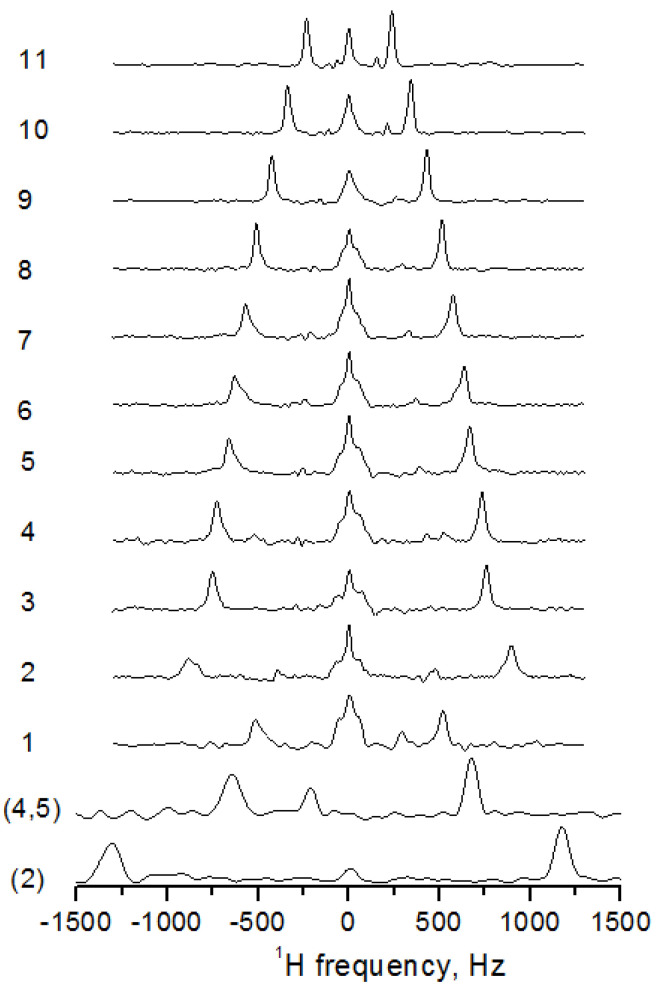
Dipolar cross section along ^1^H dimension from the two-dimensional (2D) ^13^C-^1^H PDLF spectrum of C_12_C_12_imBr in smectic A phase at 97 °C. The spectra of the imidazolium carbons, C(2) and C(4,5), were recorded in a separate experiment with a larger spectral width in the proton dimension and with a decoupling frequency optimized for imidazolium protons.

^13^C-^1^H couplings were obtained from 2D proton detected/encoded local field (PDLF) spectra of aligned mesophases. A PDLF spectrum is governed by a simple two-spin interaction and thus presents a superposition of dipolar doublets [18,28]. The cross sections from the PDLF spectrum of C_12_C_12_imBr recorded at 97 °C are shown in Figure 3. The splitting Δν observed in the PDLF experiment contributed by residual dipolar coupling $d_{CH}$ and isotropic indirect spin coupling $J_{CH}$

(1) $$\Delta \nu =k(2d_{CH}+J_{CH})$$

(*k* = 0.418 ± 0.002 is an experimentally-calibrated scaling factor of the BLEW-48 decoupling sequence [29]). The value of the coupling constant $J_{CH}$ is obtained from the ^13^C NMR spectrum in the isotropic phase and its sign is known to be positive [30].

**Figure 4:**
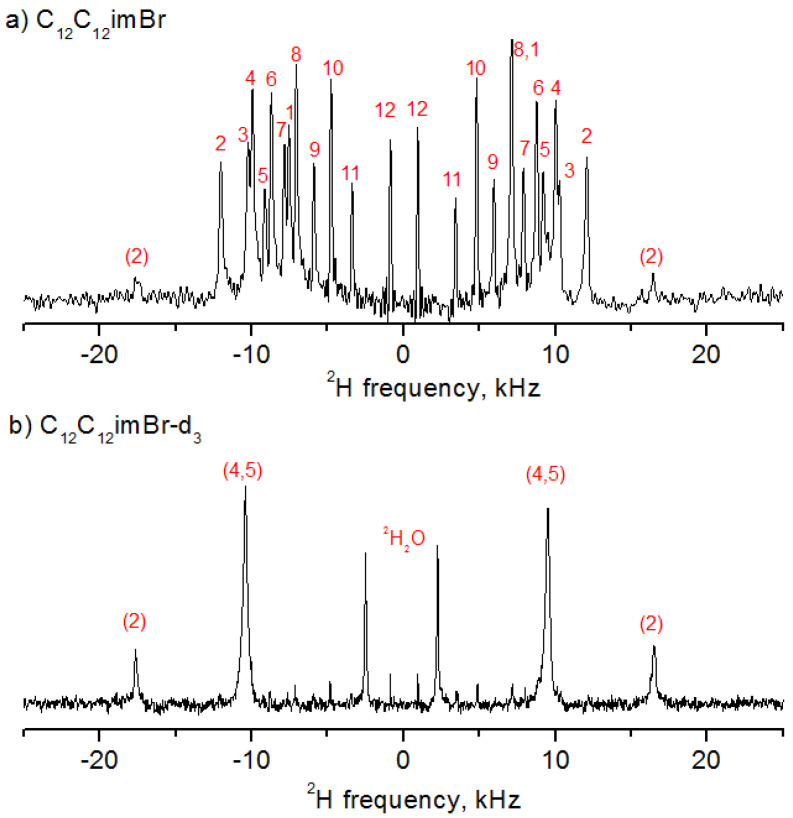
(**a**) Natural abundance deuterium (NAD) NMR spectrum of C_12_C_12_imBr at 102 °C. The spectrum resulted from the accumulation of 380 k scans during 32 h of experimental time. (**b**) ^2^H NMR spectrum of C_12_C_12_imBr-d_3_ sample enriched with ^2^H in the imidazolium ring at 102 °C. About 3 weight percent of the 90% deuterium-labelled material was added to the C_12_C_12_imBr sample with natural isotopic abundance.

Since the sign of the splitting Δν was unavailable from intrinsically symmetric shapes of dipolar spectra, additional information was required to unambiguously determine the value of dipolar coupling $d_{CH}$ from Equation (1). For the chain carbons, assuming average orientation of the molecular axis approximately parallel to the director, the value $d_{CH}$ was expected to be negative. To verify this assumption, we recorded NAD NMR spectra (Figure 4a) and compared the quadrupolar splittings $\Delta \nu_{Q}$ to the corresponding value $d_{CH}$ calculated from Equation (1). The spectrum assignment of the quadrupolar doublets was assisted by the comparison to the ^2^H NMR spectrum of the deuterium-labelled sample C_12_C_12_imBr-d_3_ (Figure 4b). For the aliphatic sites, the ratio of coupling constants $\Delta \nu_{Q}/d_{CH}\approx 11.7$ is predicted [31]. This condition could be satisfied only by assuming negative signs of $d_{CH}$ in Equation (1), thus resolving the ambiguity of the $d_{CH}$ values for the chain carbons (Table S2 in Supplementary Materials).

The same approach is not applicable to determine the signs of the couplings in the imidazolium ring because of the large variation of the quadrupolar coupling constant $\chi_{Q}$ of the aromatic sites, which participate in hydrogen bonding [32]. Therefore, we applied a method to relate signs of *J*-coupling and dipolar coupling based on off-magic angle sample spinning (off-MAS). In this technique, ^13^C spectra without proton decoupling are recorded in the samples spinning at angles close to the magic angle. Observed spectral splitting is determined by the combined effect of *J*-coupling and residual dipolar coupling. From the change of the spectral splitting depending on the spinning angle, the relative signs of *J* and dipolar couplings can be established [33]. The off-MAS spectra, shown in the Supplementary Materials, validated the negative signs of dipolar couplings obtained in the PDLF spectra for the imidazolium sites. Also, the signs for some aliphatic sites resolved in the MAS spectra were confirmed and agreed with those derived from the comparison of the dipolar and NAD spectra.

Combined molecular re-orientational motion and local conformational dynamics can be quantitatively characterized by the local C-H bond order parameters $S_{CH}=\langle 3\cos\theta_{PN}-1\rangle /2$, with $\theta_{PN}$ describing the angle between bond vector (principal frame *P*) and average molecular alignment (director frame *N*). Parameters $S_{CH}$ were estimated from Equation (2):

(2) $$d_{CH}=b_{CH}\;S_{CH}\;P_{2}(\cos\theta_{NL})=-0.5\;b_{CH}\;S_{CH}$$

where $P_{2}(\cos\theta_{NL})=(3\cos^{2}\theta_{NL}-1)/2$ and $\theta_{NL}=90{}^{\circ}$ is the angle between the director and the magnetic field vector. Any possible contribution from the biaxiality term (see below) is neglected in Equation (2). The accepted values for the dipolar coupling constants in the principal frame $b_{CH}=-(\mu_{0}/8\pi^{2})(\gamma_{H}\gamma_{C}\hbar /r_{CH}^{3})$, with account for vibration effects, are −21.5 kHz and −22 kHz for aliphatic and aromatic sites, respectively [34,35].

### 2.2. Alkyl Chain Local Order

**Figure 5:**
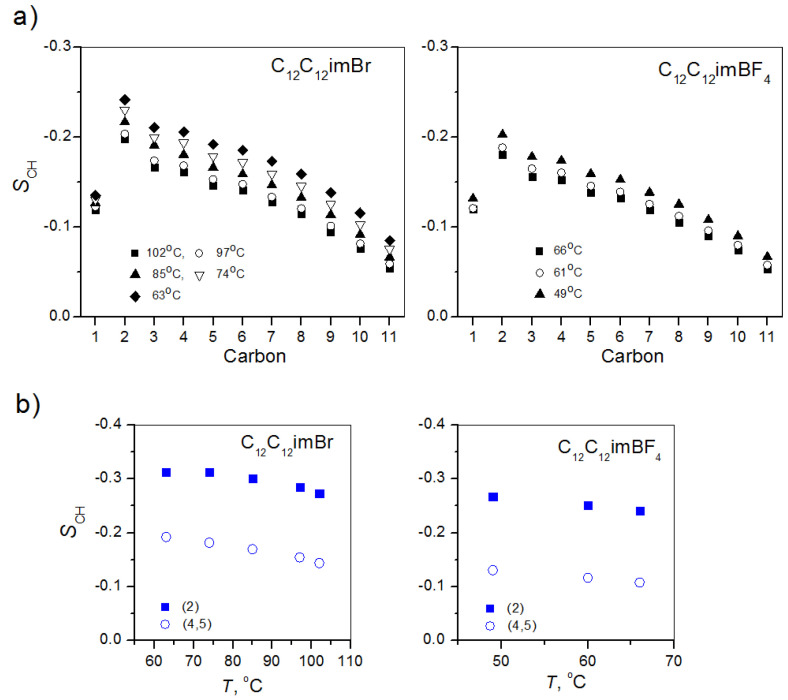
(**a**) Bond order parameter profiles of C_12_C_12_imBr (**left**) and C_12_C_12_imBF_4_ (**right**) in smectic A phase at different temperatures. (**b**) Temperature dependence of the C-H bond order parameter of imidazolium carbons in C_12_C_12_imBr (**left**) and C_12_C_12_imBF_4_ (**right**). Bond order parameters are presented in the director reference frame.

The dependencies of $S_{CH}$ values on the C-H bond position along the alkyl chains, so-called order parameter profiles, at selected temperatures are shown in Figure 5a. As expected, at decreasing temperatures the chain ordering increased. Generally, segmental mobility reduced gradually towards the chain terminal methyl. The exception was the significantly lower $S_{CH}$ value for the first methylene group. This is due to the particular conformation of the chains controlled by the alignment of the imidazolium ring, as discussed below.

Overall, order parameters are higher compared to the single-chain analogous ILCs C_12_mimBr and C_12_mimBF_4_ [21]. Generally, this is expected due to: (i) larger molecular size and (ii) increased contribution from van der Waals forces for the mesophase stabilization compared to electrostatic interaction. The order parameters are higher for Br salt compared to BF_4_ salt, in line with the trend found in single chain analogues. This behavior points to the considerable contribution of hydrogen bond interactions. We have recently shown for single chain imidazolium based ILCs that the order parameter is increasing in the anion sequence BF_4_^–^ < I^–^ < Br^–^ < Cl^–^, in accordance with increasing ability of the anion to build hydrogen bonds [21].

### 2.3. Imidazolium Core Alignment

**Figure 6:**
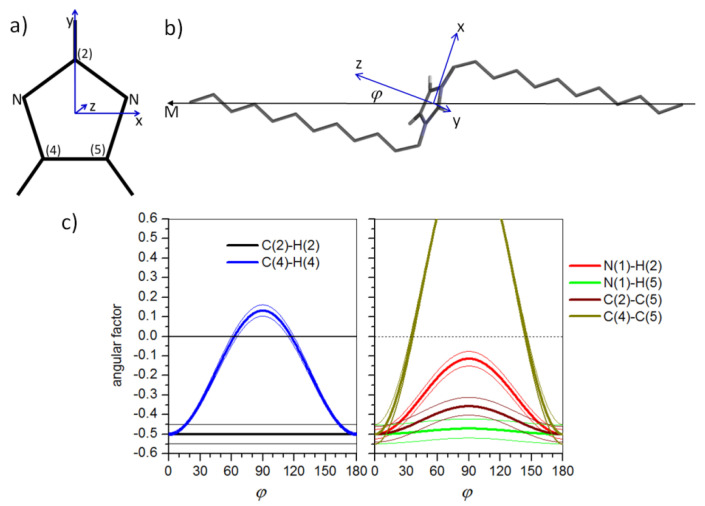
(**a**) Coordinate axis frame for the imidazolium ring. (**b**) Schematic illustration of the imidazolium core alignment to the cation long axis. (**c**) Dependence of the angular parameters of internuclear vectors $P=(3\cos^{2}\theta_{Z}-1)/2+\eta_{S}(\cos^{2}\theta_{X}-\cos^{2}\theta_{Y})/2$ on the tilt angle ϕ. Three sequential lines for each vector correspond to the biaxiality parameter values $\eta_{S}=(S_{xx}-S_{yy})/S_{zz}$ = −0.1, 0, and +0.1.

C-H bonds in imidazolium cores exhibited negative *S*_CH_ values, suggesting average alignment of the imidazolium plane approximately perpendicular to the long cation axis and the mesophase director. Additionally, smaller (by a factor of ~1.5–2) splittings observed for C(4,5) compared to C(2) (Figure 5b) indicated that the core plane is tilted to the molecular axis (Figure 6a,b). The experimental data were consistent with the tilt angle of the core plane around the y-axis (direction of the C(2)-H(2) bond) within the range of 30–40°. The angular dependencies for different internuclear vectors are shown in Figure 6c. In these plots, the angle ϕ = 0 corresponds to the core plane perpendicular to the long molecular axis. Furthermore, we verified that only a small inclination <10° of the core plane around the x-axis, superimposed on the tilt around the y-axis, is compatible with the experimental data. The analysis also included the effect of the potential non-zero biaxiality term $\eta_{S}=(S_{xx}-S_{yy})/S_{zz}$ (see caption to Figure 6).

**Figure 7:**
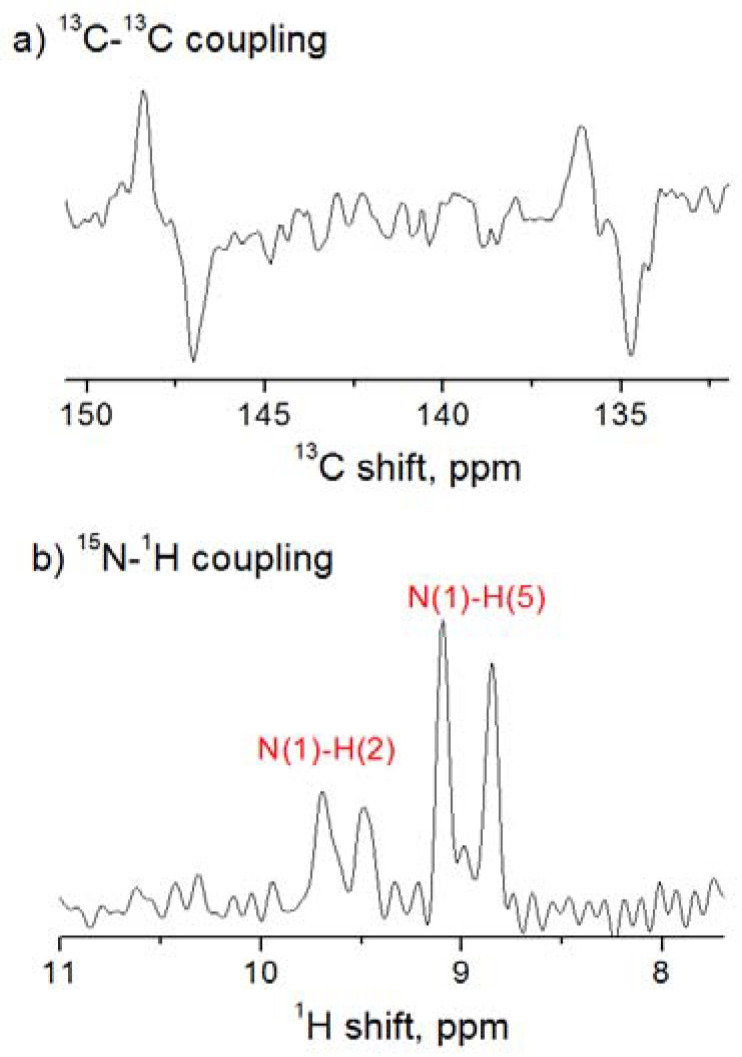
(**a**) Cross section from ^13^C-^13^C dipolar incredible natural abundance double quantum transfer experiment (dipolar-INADEQUATE) spectrum of C_12_C_12_imBr in smectic A phase at 97 °C, showing the correlation between carbon C(2) and carbons C(4) and C(5) of the imidazolium ring. From the observed antiphase splittings of 170 Hz in the dipolar doublets, the dipolar coupling constant is estimated as 85 Hz (neglecting two-bond coupling constant ^2^*J*_CC_ [30]). (**b**) ^15^N-^1^H dipolar coupling spectrum of C_12_C_12_imBr in the mesophase at 98 °C.

In an effort to obtain independent support for the above model of the cation core alignment, we measured ^13^C-^13^C and ^15^N-^1^H dipolar coupling in the imidazolium ring using a dipolar incredible natural abundance double quantum transfer experiment (dipolar-INADEQUATE) and ^15^N PDLF experiment, respectively. Coupling between C(4) and C(5) core carbons would be the most sensitive probe of the tilt angle ϕ, since the bond C(4)-C(5) makes a right angle to the y-axis (see the angular dependence in Figure 6c). In a 24-h long experiment, we could only detect correlation peaks corresponding to coupling C(2)-C(4,5) (Figure 7a) but not correlation peaks between C(4) and C(5). Note that in this experiment, the coupling within extremely rare spin pairs with a natural abundance of only 0.01% is measured. The absence of the C(4)-C(5) correlation suggests that this coupling is small, which is, in fact, consistent with the expected range of 30-40° for the tilt angle ϕ (see Figure 6c). The observed coupling ^13^C(2)-^13^C(4) of 85 Hz, and the ^15^N(1)-^1^H(2) and ^15^N(1)-^1^H(5) couplings of 123 and 148 Hz, respectively (Figure 7), are all in agreement with those derived from the model structure, within the experimental error (Table S3 in Supplementary Materials). Thus, three sets of dipolar couplings ^13^C-^1^H, ^13^C-^13^C, and ^15^N-^1^H, obtained from different experiments, are essentially compatible with the ring alignment displayed in Figure 6b. We also show below that this conformation predicts reduced average dipolar coupling and correspondingly reduced $S_{CH}$ value for the C1-H1 pair of the chains, as observed in the PDLF experiments (Figure 5a).

### 2.4. Comparison to Crystal Structures and DFT Optimized Cation Geometry

Reported crystallographic data of these and analogous symmetric long-chain compounds revealed U-shaped, V-shaped and rod-shaped cation conformations depending on anion type [3,10,11,36,37]. It has also been suggested that the general structural shape is preserved on transition to mesophase in spite of the disordered chains and fast rotational/translational dynamics [11,15,36]. Only the rod-shaped conformation is consistent with our experimental data for C_12_C_12_imBr and C_12_C_12_imBF_4_, in agreement with the reported crystal [10] or mesophase structures [11,13]. On the other hand, a V-shaped structure of the cation in mesophase has been predicted for C_12_C_12_imBr in [15].

**Figure 8:**
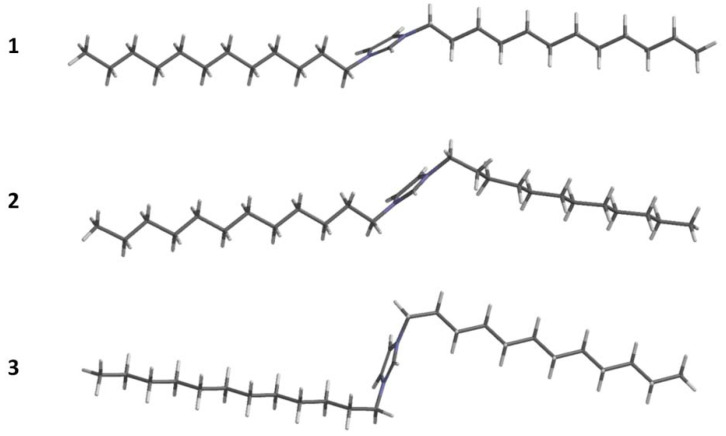
Density functional theory DFT optimized cation geometries: model with all-trans chains (**1**), with gauche bond C1-C2 in the right chain (**2**), with gauche C1-C2 bond in both chains (**3**). Molecular axis is defined as a vector connecting terminal methyl carbons. Tilt angle ϕ of the imidazolium plane (cf. Figure 7b) is, respectively, 62°, 42°, and 22°. In all models, the C(2)-H(2) vector makes an angle of ≈90° to the cation axis.

In the mesophase, where the alkyl chains are dynamically disordered, the tilt angle of the core plane is affected mainly by the conformation of the chain segments in the vicinity of the core. By examining the reported crystallographic data [3,10,37,38] and density functional theory (DFT) optimized geometries (Figure 8) we concluded that the trans conformation for these segments led to too large of a tilt angle for the core plane (ϕ *>* 60°), incompatible with the values and signs of the experimental dipolar coupling constants in the imidazolium core. On the other hand, the structures exhibiting gauche-like conformation of the first chain segments displayed a smaller tilt angle in better agreement with the experimental results; the relative magnitudes and signs of the coupling constants are correctly predicted. Figure 8 shows the DFT-optimized cation structures (in vacuo) with the single-gauche bond in one or both chains in comparison to the conformation with all-trans chain. The tilt angle of the imidazolium plane is calculated to 42° and 22° for conformations 2 and 3, respectively.

Since the model structures did not account for the conformational dynamics, they can only be used to compare local order parameters for the rigid central part and also to estimate the C-H bond orientation of the C1 site having limited motional freedom. It turns out that for the structures with gauche bonds, this estimate is in comparatively good agreement with the experimental data, at least when the signs and relative magnitudes of the dipolar couplings are concerned. Particularly, significantly decreased coupling for the C1 carbon compared to that in the next chain segments is predicted in agreement with the experimental *S*_CH_ profiles in Figure 5a. The calculated angular factor for the C1-H1 bond (average value of four C-H vectors in two methylene groups) is −0.33 and −0.18 for structures 2 and 3, respectively, while it is about −0.48 for the rest of chain segments. In contrast, the all-trans conformation predicts equal angular parameters of about −0.5 for all C-H bonds in the chains. Note that in the previous studies of ILCs with single-chain imidazolium cations, dominant all-trans chain conformation has been proven [20,21].

### 2.5. Order Parameters

Based on the working model of the imidazolium ring alignment to molecular axis, we are now in a position to estimate the cation orientational order parameter value *S*. Since the C(2)-H(2) bond in the imidazolium ring spans the angle ≈90° with the cation axis, the molecular order parameter was calculated as $S=S_{C(2)H(2)}\;/(-0.5)$. Using data from Figure 5b, the order parameter was found in the range of 0.54–0.61 and 0.48–0.53 for C_12_C_12_imBr and C_12_C_12_imBF_4_, respectively. While the obtained absolute values of the order parameters *S* are somewhat uncertain, the relative variation is robust and less sensitive to model details.

The order parameter obtained in symmetrically-substituted ILCs is higher compared to the single chain analogues [19,20,21]. In ionic liquid crystals, electrostatic and hydrogen bonding in ionic sublayer and van der Waals forces of the apolar domains are the major interactions contributing to mesophase stabilization. In the double chain ILCs, the relative contribution of van der Waals forces increased. Similarly, to conventional molecular smectics, mesophase stabilization by van der Waals interaction requires significant orientational ordering [39]. Nevertheless, the estimated *S* values in the double-chain ILCs is lower compared to that in conventional molecular smectic A phases [39,40,41], pointing to the significant roles of electrostatic and hydrogen-bonding contributions in double-chain ionic liquid crystals.

## 3. Materials and Methods

### 3.1. Synthesis

Two symmetric imidazolium-based ionic liquid crystals, bis-1,3-dodecyl imidazolium bromide (C_12_C_12_imBr) and tetrafluoroborate (C_12_C_12_imBF_4_), were synthesized following the reported procedures [13,16,42]. The compounds 1-bromododecane and 1-dodecylimidazole were purchased from Fluorochem Ltd, Hadfield, UK. To synthesize C_12_C_12_imBr, 1-bromododecane (1.2 mmol) was added dropwise to 1-dodecylimidazole acetonitrile solution (1 mmol) under stirring conditions in N_2_ atmosphere. The mixture was refluxed for 72 h at 70° C under the same conditions. The solution was subsequently condensed by evaporation, and the oily product was washed with diethyl ether several times. The white precipitate was purified by recrystallization in an ethanol/diethyl ether mixture. The final product was dried under vacuum. To synthesize C_12_C_12_imBF_4_, ion exchange was performed by adding sodium tetrafluoroborate (1.2 mmol) to acetonitrile solution of C_12_C_12_imBr (1 mmol). The mixture was stirred at room temperature for 24 h. After filtering to remove NaBr salt, the product was condensed by evaporation and washed with diethyl ether to obtain a white solid. The product was recrystallized from an ethanol/diethyl ether mixture and dried under vacuum. The sample C_12_C_12_imBr-d_3_ deuterated in an imidazolium ring was synthesized by adapting the reported procedures [43,44]. Two grams of C_12_C_12_imBr was dissolved in 10 mL of D_2_O, and 40 mg of solid NaOH was added to the solution. The reaction mixture was left overnight at 60 °C. The solution was neutralized with hydrobromic acid and the product was washed with dichloromethane four times. The purified product was dried under high vacuum. ^1^H NMR spectrum indicated a deuteration degree of ~90%.

### 3.2. NMR Methods

Experiments were performed using a Bruker 500 Avance III spectrometer (Bruker BioSpin GmbH, Rheinstetten, Germany) at Larmor frequencies of 500.1, 125.7, 76.8, and 50.7 for ^1^H, ^13^C, ^2^H, and ^15^N, respectively. NMR spectra were recorded using a custom-modified solution state multinuclear 5 mm probe head. The ^1^H, ^13^C, ^2^H, and ^15^N 90° pulse lengths were 8, 13, 6, and 50 µs, respectively. For heteronuclear proton decoupling in the mesophase, Spinal64 sequence [45] was used with the ^1^H nutation frequency of 25 kHz. To enhance the intensity of the ^13^C signal, proton-to-carbon cross polarization (CP) was applied with nutation frequencies of about 20 kHz and contact time in the range of 3–12 ms. Dipolar ^13^C- ^1^H and ^15^N-^1^H spectra were recorded using two-dimensional PDLF NMR spectroscopy [28,46]. Proton homonuclear decoupling was achieved by BLEW-48 multiple-pulse sequence [29] with a nutation frequency of 31.2 kHz. The evolution time in indirect time domain was incremented with 192 µs in 256 steps, at each with 4 or 32 collected transients for ^13^C or ^15^N experiments, respectively. Deuterium NMR spectra were recorded by quadrupolar echo sequence with an echo delay of 30 µs and in the presence of broad-band proton decoupling. Dipolar ^13^C-^13^C spectra were recorded using the 2D dipolar INADEQUATE method [20,47]. The sample temperature was regulated with an accuracy of 0.1 °C. Decoupling powers, irradiation times, and repetition delays were adjusted to limit heating effects to <0.5 °C.

### 3.3. Computational Method

Density functional theory (DFT) computational analysis of the C_12_C_12_im cation was performed using the Spartan’18 program [48]. Several conformers were examined distinguished by trans or gauche conformation of the first segments of the alkyl chain. Geometry optimization was performed for isolated cations (in vacuo) with B3LYP/6-311++G** theory level.

## 4. Conclusions

In this work, we have investigated the conformation and dynamics of symmetric double-chain cations in smectic ionic liquid-crystalline phase using multinuclear solid-state NMR spectroscopy. Molecular conformation was characterized in terms of local bond order parameters. The obtained experimental data are consistent with the model of rod-shaped cations with the two chains aligned in opposite directions outwards from the imidazolium core. The average alignment of the core plane and the restricted conformations of the chain segments in the vicinity of the core were found to be different from that in single chain analogues. Quantum-chemical DFT analysis supported the experimental findings. Besides, the overall rod-shaped cation conformation in mesophase was compatible with reported crystallographic structures.

The orientational order parameter *S*~0.5–0.6 of double chain ILCs was higher than that of corresponding single-chain counterparts. This was consistent with the enhanced contribution of van der Waals forces to the stabilization of smectic layers. Increased orientational order for the material with Br^−^ counterions, which exhibit lower charge delocalization (smaller ionic radius) and higher ability to hydrogen bond as compared to that of BF_4_^−^, also indicated a non-negligible influence of electrostatic and hydrogen bond interactions. Nanoscale segregation of polar and apolar domains induced by these forces contributed to layered structure stabilization. While in single-chain ILCs, a fragile equilibrium between the interactions in polar and hydrophobic sublayers resulted in low order parameter of *S*~0.3, in the double-chain analogues, the dominant van der Waals forces enhance the orientational ordering.

This study represents a step toward elucidating the complex ion dynamics and the roles of different intermolecular forces in the formation and stabilization of mesophase in mesogenic ILs. A better understanding of the interaction between the ionic constituents should provide important feedback for the improved and rational design of new functional materials tailored for specific applications. It is anticipated that our approach will be equally efficient for other types of ILCs. In future studies, it would be interesting to investigate cation dynamics and orientational order in symmetrical imidazolium-based di-cationic mesogenic salts, which offer a greater variability in their properties.

## Supplementary Materials

Supplementary materials can be found at https://www.mdpi.com/1422-0067/21/14/5024/s1.

## Abbreviations

C_12_C_12_imBr	bis-1,3-dodecyl imidazolium bromide
C_12_C_12_imBF_4_	bis-1,3-dodecyl imidazolium tetrafluoroborate
CS	chemical shift
DFT	density functional theory
ILs	ionic liquids
ILCs	ionic liquid crystals
INADEQUATE	incredible natural abundance double quantum transfer experiment
NAD	natural abundance deuterium (spectroscopy)
off-MAS	off-magic angle sample spinning
PDLF	proton detected/encoded local field (spectroscopy)
