# Supplementary material for: NMR Spectroscopic Studies of Cation Dynamics in Symmetrically-Substituted Imidazolium-Based Ionic Liquid Crystals
Source: Int J Mol Sci. 2020 Jul 16;21(14):5024. doi: 10.3390/ijms21145024 (PMC7404116; doi:10.3390/ijms21145024)
Supplement: Supplementary file 1 [file ijms-21-05024-s001.pdf]

Supplementary Materials

Table S1. Phase transition temperatures in $C_{12}C_{12}imX$ samples (X = BF_4, Br).

Sample	$T_{Cr\rightarrow Sm}$ °C	$T_{Iso\rightarrow Sm}$ °C	Water Content mol%
$C_{12}C_{12}imBF_4$	53	71	0
$C_{12}C_{12}imBr$	50	107	7
3 wt% $C_{12}C_{12}imBr$-d_3 in $C_{12}C_{12}imBr$	50	107	0.6 (D_2O)<br>6 (H_2O)

Table S2. Dipolar couplings d_{CH}, quadrupolar splittings Q, and their ratios in the alkyl chain of the $C_{12}C_{12}imBr$ cation in smectic A phase at 102 °C.

Carbon	d_{CH}, Hz	$\|Q\|$, Hz	$\|Q/d_{CH}\|$
C1	−1284	14750	11,5
C2	−2132	24280	11,4
C3	−1790	20650	11,5
C4	−1730	20100	11,6
C5	−1576	18440	11,7
C6	−1517	17600	11,6
C7	−1376	15870	11,5
C8	−1231	14290	11,6
C9	−1017	11940	11,7
C10	−818	9677	11,8
C11	−586	6900	11,8

Table S3. Calculated dipolar coupling, $d = b \cdot P_2^{(PM)} \cdot S \cdot (-0.5)$, for imidazolium ring inclined at the angle ϕ = 35° to the molecular axis. Order parameter S = 0.55. Dipolar coupling constants in the principal frame of the interaction, $b_{SI} = -(\mu_0 / 8\pi^2)(\gamma_S \gamma_I \hbar / r_{SI}^3)$, were calculated with the inter-atomic distances obtained from DFT-optimized cation geometry.

Pair	b, Hz	$P_2^{(PM)}$	d, Hz calculated	d, Hz experimental
C(2)-H(2)	−22000[a)]	−0,500	−3025	−3000
C(4)-H(4)	−22000[a)]	−0,292	−1767	−1600
C(2)-C(4)	−713	−0,453	−88,8	−85
C(4)-C(5)	−3020	−0,007	−5,8	~0
N(1)-H(2)	1225	−0,373	125,7	123
N(1)-H(5)	1208	−0,490	162,8	148

[a)] Accepted values for the dipolar couplings with account for vibration effects.

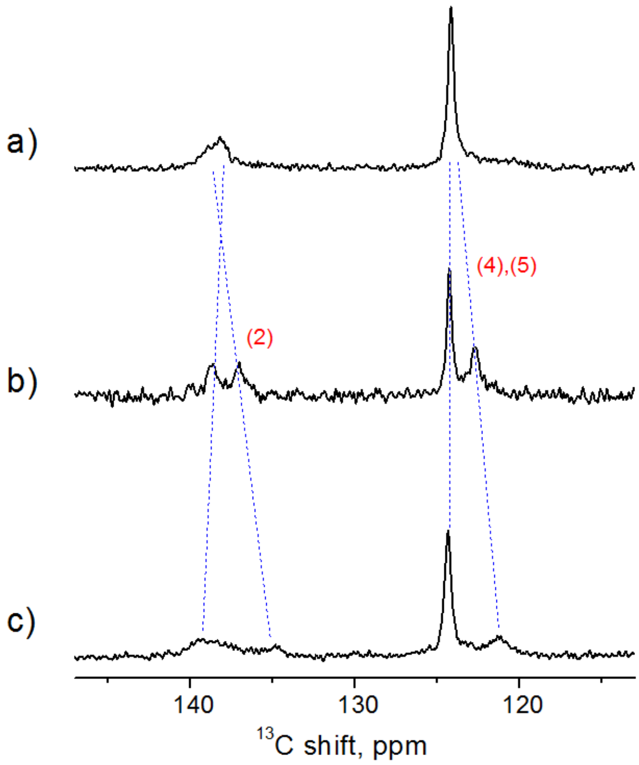

Figure S1. ^{13}C spectra of the sample $C_{12}C_{12}$imBr spinning at different angles. The sample was in the smectic A phase at 60 °C. Spectra were recorded at spinning speed of 10 kHz and without proton decoupling. Spinning angles were 52.1° (**a**), 54.7° (magic angle) (**b**), and 58.0° (**c**). Only signals of imidazolium ring are shown. Doublets due to ^{13}C-^{1}H J-coupling are observed in the spectrum (**b**). In off-MAS spectra (**a**) and (**c**), powder line-shapes are observed due to contributions of the ^{13}C residual chemical shift anisotropy and ^{13}C-^{1}H dipolar interactions.
